# Hospital Contracts and Prices—II

**Published:** 1910-04-16

**Authors:** 


### Special Departmental Article.

### HOSPITAL CONTRACTS AND PRICES—II.

Contributions and questions on matters affecting this department of an institution are invited.

In our former article we dealt with the questions of tender forms and the most favourable periods of the year in which to enter into contracts for supplies of provisions. In the present article we propose to consider the acceptance of tenders and to refer to the prices at which many of the most important articles of food can be obtained at the present time.

A few years ago it was customary for hospitals to use nothing but English meat, but the low price and excellent quality of American chilled beef, New Zealand mutton, and Canterbury lamb have revolutionised the custom, and comparatively few hospitals now purchase English meat only.

The finest chilled meat can now be purchased at the following prices:—

Finest United States Chilled Beef.

Hind quarters, 5¼d. per lb.
Fore quarters, 4d. per lb.
Rounds (without bones, legs and flanks, with udder fat taken off), 6⅝d. per lb.
Sirloins, free from suet; ribs, best end only, both cut short (not longer than 12 inches), 7d. per lb.
Neck, without bone, 3⅝d. per lb.
Shins and legs, London cut, 3d. per lb.
Best beef steak, 8d. per lb.
Suet, best beef kidney, 3d. per lb.
Beef bones, 1s. per score.

Finest New Zealand Mutton.

Whole carcase, 4½d. per lb.
Loins, trimmed and free from inside fat, 5d. per lb.
Legs, 5d. per lb.

Finest Canterbury Lamb.

Whole carcase, 4⅝d. per lb.

Hind quarters, 6d.

Finest English Veal.

Fillet, without bone, 7½d. per lb.

Finest English Pork.

Legs and loins, 7½d. per lb.

Chilled meats require to be dealt with carefully. They must not be put into the oven in their frozen state, or they will not give satisfaction. If allowed to hang in a cool, dry, airy store for at least twenty-four hours after they are removed from the cold chamber, all frost disappears, the meat cooks well, is tender and juicy, and, especially in the case of beef, it would be difficult to find an English joint to excel it in flavour and tenderness. A few months ago the joints enumerated could be purchased at considerably lower rates, and there is little doubt that the old favourable prices will again be paid in the near future.

It is astonishing how the price of fish varies in different towns. In the Midlands it is a distinct advantage to procure it direct from Grimsby. If a reasonable quantity is required it can at the present time be obtained delivered free daily at the following rates, the quality in each instance being of the best:—

Codfish, without head and flaps, cleaned, ready for cooking, 1⅝d. per lb.

Hake, 2d. per lb. Halibut, 5d. per lb.

Plaice, 3d. per lb. Whiting, 2d. per lb.

Haddock, 1½d. per lb.

Other items of provisions can be purchased at the following rates:—

Chickens, weighing 1¾ lb. each, dressed ready for cooking, 1s. 8d. each.
Finest Danish butter, 118s. per cwt.
Finest fresh Irish eggs, 15-16 lb. per 120, 7s. per 120.
Milk, guaranteed free from tuberculosis, 8½d. per Imperial gallon.
Bread, 1s. 5d. per stone of 16 lb.
Finest Danish smoked bacon, full sides 8d. per lb.
Best Irish roll and smoked hams, 8d. per lb.
There is a good deal of variation in the prices paid by different hospitals for tea, but, bought by the chest, a very good tea can be purchased for 1s. 2d. per lb.
An excellent coffee in the berry, fresh roasted, can be purchased for 1s. per lb.
Granulated sugars, from 17s. to 18s. per cwt.
Rice (Rangoon), 11s. per cwt.
Potatoes this year are particularly cheap; 6s. 6d. to 8s. per load of 252 lb. cannot be considered dear at this time of the year for really good potatoes.

Malt liquors are very little used in the best hospitals nowadays, and a very good light ale can be purchased at 10d. per gallon. Mineral-waters should not be purchased to any great extent. If the consumption is large a soda-water machine and a few gross of syphons are excellent investments, enabling soda water to be manufactured at a few pence per dozen syphons, because there is always a porter or lad about who can fit in the work of charging the syphons with his ordinary duties, and the institution pays nothing additional for labour.

In addition to care in purchasing, keen supervision of diets is necessary to keep down expenditure. It is so easy to keep patients upon expensive diets after the necessity for special feeding has disappeared. A good ward sister by a judicious word to a resident house surgeon or house physician can assist materially in preventing extravagance and unnecessary dieting of patients, and a skilled administrator by a comparison of the cost per head of patients' diets in different wards can ascertain from week to week if any extravagant dieting is taking place in any part of his hospital.

